# Does Reduction of Number of Intradetrusor Injection Sites of *aboBoNTA* (Dysport^®^) Impact Efficacy and Safety in a Rat Model of Neurogenic Detrusor Overactivity?

**DOI:** 10.3390/toxins7124896

**Published:** 2015-12-17

**Authors:** Amélie Huynh Le Maux, Bernadette Pignol, Delphine Behr-Roussel, Jean-Luc Blachon, Pierre-Etienne Chabrier, Sandrine Compagnie, Philippe Picaut, Jacques Bernabé, François Giuliano, Pierre Denys

**Affiliations:** 1 Pelvipharm, 2 avenue de la source de la Bièvre, Bâtiment Simone Veil, Université de Versailles-Saint-Quentin-en-Yvelines, Montigny-le-Bretonneux 78180, France; a.lemaux@pelvipharm.com (A.H.L.M.); s.compagnie@pelvipharm.com (S.C.); j.bernabe@pelvipharm.com (J.B.); 2 Unité de Formation et de Recherche des Sciences de la Santé, Université de Versailles-Saint-Quentin-en-Yvelines, Institut National de la Santé Et de la Recherche Médicale U1179, Montigny-le-Bretonneux 78180, France; francois.giuliano@uvsq.fr (F.G.); pierre.denys@rpc.aphp.fr (P.D.); 3 IPSEN Innovation, 5 avenue du Canada, Les Ulis 91140, France; bernadette.pignol@ipsen.com (B.P.); jean-luc.blachon@ipsen.com (J.-L.B.); pierre-et.chabrier@ipsen.com (P.-E.C.); philippe.picaut@ipsen.com (P.P.); 4 Assistance Publique-Hôpitaux de Paris, Neuro-Uro-Andrology, Raymond Poincaré Hospital, Department of Physical Medicine and Rehabilitation, Garches 92380, France

**Keywords:** *abobotulinumtoxinA*, injection procedure, injection site number, neurogenic detrusor overactivity, spinal-cord injury

## Abstract

Intradetrusor injections of *Botulinum toxin A*—currently onabotulinumtoxinA—is registered as a second-line treatment to treat neurogenic detrusor overactivity (NDO). The common clinical practice is 30 × 1 mL injections in the detrusor; however, protocols remain variable and standardization is warranted. The effect of reducing the number of injection sites of Dysport^®^ abobotulinumtoxinA (*aboBoNTA*) was assessed in the spinal cord-injured rat (SCI). Nineteen days post-spinalization, female rats received intradetrusor injections of saline or *aboBoNTA* 22.5 U distributed among four or eight sites. Two days after injection, continuous cystometry was performed in conscious rats. Efficacy of *aboBoNTA* 22.5 U was assessed *versus* aggregated saline groups on clinically-relevant parameters: maximal pressure, bladder capacity, compliance, voiding efficiency, as well as amplitude, frequency, and volume threshold for nonvoiding contractions (NVC). *AboBoNTA* 22.5 U significantly decreased maximal pressure, without affecting voiding efficiency. Injected in four sites, *aboBoNTA* significantly increased bladder capacity and compliance while only the latter when in eight sites. *AboBoNTA* significantly reduced NVC frequency and amplitude. This preclinical investigation showed similar inhibiting effects of *aboBoNTA* despite the number of sites reduction. Further studies are warranted to optimize dosing schemes to improve the risk-benefit ratio of *BoNTA*-based treatment modalities for NDO and further idiopathic overactive bladder.

## 1. Introduction

Intradetrusor injections of *Botulinum toxin A* (*BoNTA*), *i.e.*, *onabotulinumtoxin A* (*onaBoNTA*), first tested in spinal cord-injured (SCI) patients in 2000 [1], is approved as of 2011 by the US Food and Drug Administration and European Union as a second-line treatment to treat neurogenic detrusor overactivity (NDO) in humans.

*BoNTA* blocks the release of acetylcholine (ACh) and norepinephrine from autonomic nerves in the rat bladder [2], of adenosine triphosphate (ATP) from bladder urothelium of chronic SCI rats [3] and the excretion of neuronal growth factor (NGF) in NDO patients [4]. *BoNTA* also inhibits neurally-evoked bladder contractions [5] and activation of bladder afferents [6,7,8,9]. Although the efficacy of *BoNTA* is now well-established [10,11,12,13,14,15], dosing and injection schemes, including their risk-benefit ratio evaluation, can still be questioned.

The majority of clinical published data relates to *onaBoNTA* (Botox^®^) and, to a lesser extent, to *abobotulinumtoxinA* (*aboBoNTA*, Dysport^®^). The potency of these two formulations is different and doses cannot be interchanged. Nonetheless, intradetrusor injections in NDO patients of either formulations result in an increase in maximal bladder capacity and compliance, associated with decreased maximum detrusor voiding and filling pressures, and attenuation of uninhibited detrusor contractions (UDC) [10,11,12,13,14,15,16,17].

Various intradetrusor injections protocols have been described differing in dosage, dilution, as well as in number and location of injection sites. It is now of importance to harmonize clinical procedures in order to optimize dosing schemes. Several questions remain to be answered, especially the influence of variation of injection sites on urodynamic outcomes. This study is the first preclinical investigation evaluating the efficacy of *aboBoNTA* intradetrusor injections when reducing the number of injection sites. This comparison was performed using clinically relevant urodynamic parameters assessed by continuous cystometry in the SCI rat model [18,19], displaying a voiding pattern comparable to that of neurogenic patients [20], that has already been validated in-house for investigations on *BoNTA* [21].

## 2. Results

### 2.1. Effect of Intradetrusor Injections of aboBoNT-A 22.5U in Four or Eight Sites in Rats 19 Days after SCI-Induced NDO

**Figure 1:**
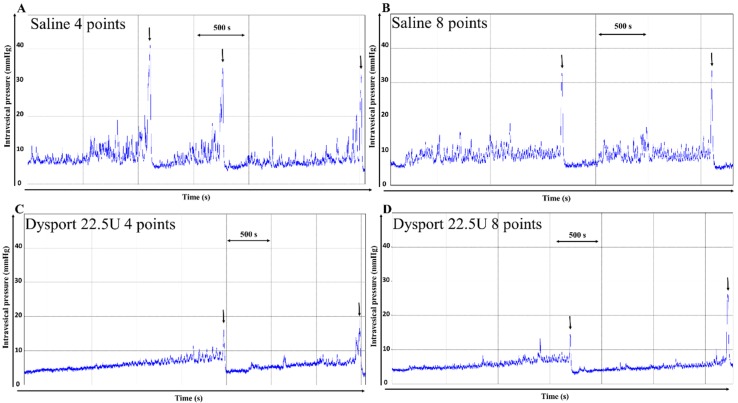
Representative cystometrograms. The urodynamic evaluation were performed at D21 post-spinalization, *i.e.*, two days after intradetrusor injection of (**A**) saline 4 pts, (**B**) saline 8 pts, (**C**) *aboBoNTA* 8 pts or (**D**) *aboBoNTA* 22.5 U. Arrows correspond to a voiding contraction.

As data were not significantly different for all urodynamic parameters, S-4 sites and S-8 sites groups were aggregated (ns, *p* > 0.05; Table 1). *AboBoNTA* 22.5 U administered in either four or eight sites significantly decreased maximal pressure (*p* < 0.001 for both sites number; Figure 1C,D and Table 1) when compared with aggregated controls (Figure 1A,B and Table 1) without affecting voiding efficiency (*p* > 0.05 for both; Figure 1C,D and Table 1). When injected in four sites, *aboBoNTA* 22.5 U significantly increased the infused volume, an index of bladder capacity, and compliance (*p* < 0.01; Figure 1C,D and Table 1), this latter being also improved with injection in eight sites albeit not significantly (*p* = 0.0584; Figure 1D and Table 1).

*AboBoNTA* 22.5U significantly decreased the amplitude of NVC when injected in four and eight sites (*p* < 0.05 and *p* < 0.01 respectively; Figure 1C,D and Table 1). NVCs’ frequency was also decreased, although not significantly for both four and eight sites (*p* = 0.0525 and *p* = 0.0772, respectively; Figure 1C,D and Table 1). No effect was observed on the volume threshold (*p* > 0.05; Figure 1C,D and Table 1).

**Table 1:** Effect of aboBoNTA *versus* aggregated saline on urodynamic parameters.

Treatment	Saline (Aggregated)		*AboBoNTA* 22.5U		Corresponding Clinically Relevant Parameters
Number of Injection Sites	4	8	4	8	
**Maximal amplitude, mmHg**	30.3 ± 1.4	30.0 ± 1.9	**23.2 ± 1.2 ^###^**	**22.7 ± 0.9 ^###^**	Maximal pressure at contraction (P Max)
**Voiding efficiency, %**	87.2 ± 4.9	87.3 ± 3.9	90.9 ± 4.7	88.2 ± 4.6	Post-void residual volume in bladder
**Infused volume, µL (Bladder capacity)**	801.1 ± 45.6	898.9 ± 69.6	**1186.6 ± 101.2 ^##^**	999.2 ± 81.9	bladder storage capacity
**Compliance, mL/mmHg**	0.13 ± 0.02	0.19 ± 0.03	**0.25 ± 0.03 ^##^**	0.21 ± 0.02	Compliance (index, mL or cm H_2_O)
**NVC amplitude, mmHg**	5.9 ± 0.8	5.1 ± 0.4	**4.3 ± 0.3 ^#^**	**3.9 ± 0.2 ^##^**	Pressure at first involuntary contraction
**NVC frequency, nb per min**	1.3 ± 0.2	1.4 ± 0.3	0.9 ± 0.2	1.0 ± 0.2	-
**NVC volume threshold, %**	49.5 ± 8.1	40.8 ± 7.3	58.7 ± 6.1	52.0 ± 5.7	Bladder volume at first contraction

Effect of intradetrusor injections of saline or *aboBoNTA* 22.5 U injected in four or eight sites on urodynamic clinically relevant parameters evaluated by continuous cystometry in SCI rats at two days post-injection (21 days post-spinalization). Data are the mean plus or minus standard error of the mean. ANOVA between S-4 sites and S-8 sites groups: ns, *p* > 0.05. Thus, aggregation is allowed by the non-significant difference between control groups S-4 sites and S-8 sites. ANOVA *versus* aggregated controls: ^#^ *p* < 0.05, ^##^ *p* < 0.01, ^###^ *p* < 0.001.

### 2.2. Effects of Treatments on Body Weight

Before intradetrusor injections, body weight of SCI rats was similar between all experimental groups (S-4 sites: 289.2 ± 14.1 g; *aboBoNTA*-4 sites: 280.1 ± 10.9 g; S-8 sites: 285.2 ± 21.0 g; *aboBoNTA*-4 sites: 285.0 ± 12.7). Four days post-intradetrusor injections, body weight was significantly lower for *aboBoNTA*-4 sites compared to S-4 sites (237.2 ± 3.8 *versus* 279.8 ± 4.1 g, respectively, *p* < 0.001) and *aboBoNTA*-8 sites compared to S-8 sites (253.3 ± 5.5 *versus* 279.9 ± 6.0 g respectively, *p* < 0.01). There was no difference in the AUC of BWL between *aboBoNTA*-4 sites and *aboBoNTA*-8 sites groups (Table 2). Of note, one a*boBoNTA*-4 sites-treated rat died three days post-intradetrusor injection.

**Table 2:** Effect of aboBoNTA on body weight.

Treatment	*AboBoNTA* 22.5 U	
Number of injection sites	Four	Eight
**AUC BWL at day one post-injection (% × day)**	−3.4 ± 0.2	−3.4 ± 0.2
**AUC BWL from day one to day two post-injection (% × day)**	−10.3 ± 0.5	−9.7 ± 0.5
**AUC BWL from day one to day tree post-injection (% × day)**	−19.8 ± 1.2	−17.2 ± 1.3
**AUC BWL from day one to day four post-injection (% × day)**	−33.5 ± 2.1	−27.8 ± 2.3

Effect of intradetrusor injections of *aboBoNTA* 22.5 U administrated in four or eight sites on area under curve (AUC) of body weight loss (BWL, % *versus* D0). Data are the mean plus or minus standard error of the mean. Fisher’s test for variance homogeneity: ns, *p* > 0.05. Student’s *t*-test: ns, *p* > 0.05.

## 3. Discussion

This is the first preclinical investigation comparing the effect of *aboBoNTA* intradetrusor injections when reducing the number of sites for injection in the SCI rat model. *AboBoNTA* 22.5 U significantly and similarly lowered VC maximal pressure and improved compliance of the bladder, albeit not significantly when injected in eight sites. Moreover, bladder capacity was increased with four injection sites. Whatever the number of sites, the amplitude of NVC was significantly decreased. In addition, frequency of NVC was decreased although not significantly when injected in four or eight sites. Finally, a similar BWL was observed post-intradetrusor injection whatever the number of injection sites.

As the bulk of available evidence on the efficacy of *BoNTA* to treat NDO increases, the need to determine an optimal dosing and administration scheme, both cost-effective and patient-oriented, becomes more and more meaningful. These experiments were performed in a preclinical model undergoing a pathophysiological process comparable to that occurring in SCI patients and characterized by the emergence of NDO, thereby mimicking at best the voiding pattern of neurogenic patients [20]. Indeed, the SCI rat displays increased intravesical maximal pressure and occurrence of NVC during the bladder filling phase, the latter ensuing from afferent C-fibers recruitment [22]. Main considerations for the treatment of NDO are maintenance of low bladder pressures to prevent development of upper urinary tract complications and suppression of UDC, in order to improve bladder capacity, as well as to decrease urinary frequency and urgency. Therefore, the study design fit as closely as possible to the clinical *BoNTA* injection procedure and urodynamic parameters were selected to best characterize those clinically relevant in neurogenic patients (Table 1). Dosing of *aboBoNTA* was chosen according to the previous dose-escalating study *i.e.*, at a ratio 3:1 of the 7.5 U minimal effective dose (MED) of *onaBoNTA* determined in the same experimental paradigm [21]. Indeed, although they are not strictly speaking interchangeable, a 3:1 to 6:1 ratio of *aboBoNTA:onaBoNTA* is commonly reported to yield similar therapeutic effects [23,24]. Hence, intradetrusor injections of *aboBoNTA* delivering 22.5 U in total were performed in four or eight sites (1.8 and 0.9 U/µL per site, respectively).

Similarly to what is reported in NDO patients, *aboBoNTA* 22.5 U intradetrusor injections in SCI rats demonstrated an effect on both efferent and afferent neural pathways. Indeed, *BoNTA* is known to cleave synaptosomal-associated protein–molecular weight 25 kilodalton (SNAP-25) protein of the soluble *N*-ethylmaleimide sensitive factor attachment protein receptor (SNARE) complex involved in ACh exocytosis at the neuro-muscular junction [25]. Inhibition of ACh release at the presynaptic nerve terminals accounts for the effect of *BoNTA* on bladder efferent signalling, with a reduction of VC maximal pressure, as already observed in this model [2]. This also matches the decrease of maximal pressure at contraction obtained in *BoNTA*-treated NDO patients [10,11,12,14,17]. This decrease can sometimes increase post-void residual volume [10], meaning urinary retention and subsequent risk of urinary tract infection in *BoNTA*-treated patients [26]. However, such an effect on the post-void residual volume was not found since voiding efficiency remained unchanged. Furthermore, apart from its direct action at the presynaptic level, *BoNTA* also modulates bladder afferents that also express SNAP-25 [27]. BoNTA has been demonstrated to affect the release of other neurotransmitters in bladder tissue such as ATP, substance P, calcitonine gene-relatide peptide (CGRP), and glutamate [3,6,27]. An additional impact of *BoNTA* on bladder afferent pathways is the decrease of sensory receptor expression levels like P2X3 or the transient receptor potential vanilloid subfamily 1 (TRPV1) [28], as well as NGF excretion [29,30]. Here, the attenuation of NVC, as well as the increased bladder compliance and capacity, are in accordance with the inhibition of bladder sensory afferent signalling by *BoNTA* in SCI rats [31]. Indeed, reduction of NVC following *onaBoNTA* intradetrusor injections has already been reported [2,19], as well as a decrease in frequency and amplitude of NVC following *aboBoNTA* treatment [21], although nature and concentration of injected compound were different from the present study. Similarly, *BoNTA* injections attenuate UDC in neurogenic patients, with their pressure at first involuntary contraction being decreased [14,30] and detrusor volume at first contraction increased [13,30]. Improvement of bladder storage capacity [10,11,12,14,17] and compliance [14] has also been reported in SCI patients after *BoNTA* treatment. Thus, all in all, the efficacy on clinically-relevant urodynamic parameters is similar for four and eight sites of injection at 22.5 U total dose of *aboBoNTA* in the SCI rat model.

From a clinical point of view, higher local diffusion of toxin is awaited with higher total volume and number of injection sites *i.e.*, lower concentrated *BoNTA* at each site, as reported in the unique clinical study on toxin distribution by magnet resonance imaging after injection in the bladder wall [32]. Likewise, when functionally studying toxin diffusion in the bladder of guinea pigs, a single injection of BoNTA 2U in 20 µL of saline induces increased spread in bladder from the injection site than in 2 µL [33]. However, since the first *BoNTA* injection description for NDO treatment [1], injections from 10 to 50 sites were tested, and the only one direct comparative study reported for 30 *versus* 10 injection sites concluded that lowering the number of injection sites did not impair efficacy [34]. As in the present study, where the main clinically-relevant parameters (maximal pressure and NVC) were similarly impacted with *BoNTA* in four or eight injections sites. An additional improvement of infused volume (index of bladder capacity) and compliance was even observed with four injection sites. Thus, *BoNTA* diffusion, and associated efficacy on NDO, was not impaired in the rat bladder. Concordantly, Liao *et al.* recently observed that similar therapeutics effects were produced by injection at 10, 20, and 40 sites in patients with refractory detrusor overactivity [35]. Finally, study results of a phase IIa clinical trial with NDO patients (ClinicalTrials.gov Identifier: NCT01357980) revealed a reduction of urinary incontinence episodes, a main symptomatic features investigated in the clinic, as well as of maximum detrusor pressure, and an increase of maximum cystometric capacity with *aboBoNTA* 750 U injected in 15 or 30 sites.

Diminishing the number of sites is still a matter of debate as, despite allowing a faster and consequent less painful injection procedure, it implies a careful investigation of safety aspects to avoid deleterious consequences. Indeed, with fewer injected sites *i.e.*, greater concentrations per site, the risk for greater distal and/or systemic effects is increased due to unwanted toxin spread out of the bladder wall. Indeed, adverse events (AE) are closely related to toxin propensity to spread, diffuse and migrate, along with dilution and total volume injected [36]. Investigations of the link between the injected concentration per site, *BoNTA* distribution beyond its original injected site, and undesired side-effects are scarce. The only direct clinical comparative study reported that reducing the number of injection sites from 30 to 10 did not impair safety [34]. However, injection of the same dosing with a lower number of injection sites, meaning a higher concentration per injection site as in the present study in SCI rat, could cause a higher toxin leakage out of the detrusor [32]. In the current study, both treatment regimen affected rats’ body weight to the same extent, although one death was reported for the rats treated with *aboBoNTA* 22.5 U at four sites of injection. Systemic AE after *BoNTA* injection have already been measured by body weight changes in mice [37] and such an effect on body weight has already been reported with *onaBoNTA* in rats, although in a different model and with a different treatment paradigm [38]. Additionally, in clinical studies, decreasing concentration per site appears to play a role in the prevention of systemic side effects, with no more side-effects reported, either by reducing the total dose [39] or by increasing the number of injection sites [40]. Thus, an excess of toxin at each injection site might spread, thereby increasing AE [41]. Furthermore, a technical incident at one site could further increment deleterious consequences for patients. For example, one patient was reported with 40% of toxin found out of the detrusor due to penetration depth hitch for one out of 10 injections [32].

Drawing a parallel between clinical and preclinical results points out their similarities and further supports the use of the SCI model for evaluation of the effect of *BoNTA* intradetrusor injections [18,19,21]. One can argue that the increase in the NVC volume threshold previously observed with *aboBoNTA* 10U injected in eight sites was not reproduced, albeit using a higher dose. However, the nature of the product was different and the prior study was a dose-response-based investigation therefore built on a different statistical analysis [21]. The only limitation of the SCI rat model is that urgency and urinary incontinence, main symptomatic clinical features investigated, are not quantified in animal preclinical studies. Although the main aim of this study was to compare the therapeutic effect of *aboBoNTA* when reducing number of injection sites in the bladder, additional experimental endpoints could provide further indications as to the safety aspects. Furthermore, BWL might have been the consequence of a decrease in food and water intake due to a *BoNTA*-induced transient muscular weakness as it was already reported in patients [42]. Thus, monitoring those consumptions and evaluation of neuromuscular function by measurement of grip strength could also be informative, as well as harvesting of bladder, followed by histological assessment.

## 4. Conclusions

This preclinical study focused on a key aspect of the *BoNTA* injection procedure (*i.e.*, concentration and sites number) and is a first step for standardization of clinical protocols. The main result of the present study is that reduction by half of the number of sites for *aboBoNTA* injection did not hamper urodynamic improvements awaited in the SCI rat model. However, a higher number of sites might be more conservative from a safety perspective. Thus, additional data on the safety aspects are warranted to determine whether decreasing the number of sites allows an improvement of the benefit-risk ratio.

## 5. Experimental Section

All procedures were performed in compliance with the legislation on the use of laboratory animals (NIH publication N 85-23, revised 1996) and Animal Care Regulations in force in France as of 1988 (authorization from competent French Ministry of Agriculture—Agreement No. A78-322-3, 2013).

### 5.1. General Experimental Design

After one week of acclimation with free access to standard chow and water, female Sprague–Dawley rats (*n* = 63; 250–275 g; Janvier, France) underwent a T8-T9 spinal cord transection [21]. Four experimental groups were considered, with intradetrusor injections taking place at D19 post-spinalization: saline (NaCl 0.9%) four sites (*n* = 12; S-4 sites), saline eight sites (*n* = 11; S-8 sites), *aboBoNTA* 22.5U four sites (*n* = 20; *aboBoNTA*-4 sites) and *aboBoNTA* 22.5 U eight sites (*n* = 20; *aboBoNTA*-8 sites). Each rat was weighed daily after injections, as it was previously observed that body weight loss (BWL) peak was reached after four days [21]. Urodynamic investigation was performed at two days post-intradetrusor injections (D21 postspinalization) and rats were euthanized at four days (D23 postspinalization).

### 5.2. Saline or aboBoNTA Intradetrusor Injections

**Scheme 1:**
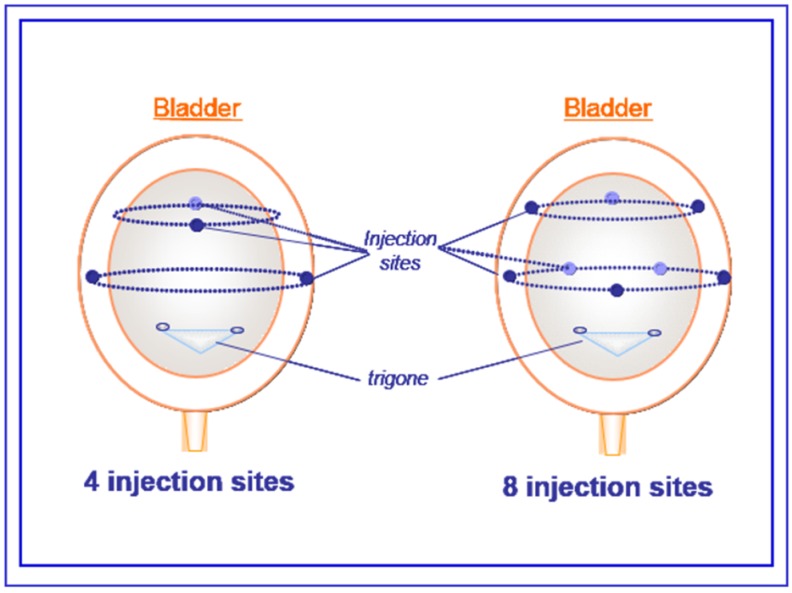
*AboBoNTA* intradetrusor injections, Schematic representation of intradetrusor injections with a needle (30-gauge) in four (total volume of 12,5 µL) or eight divided sites (total volume of 25 µL) of bladder sparing the trigone.

At 19 days post-spinalization, *i.e.*, when the NDO is well-established, bladders were exposed under isoflurane anesthesia (1.5%–2.0%) and emptied. Using a microscope, *aboBoNTA* or saline was injected into the detrusor sparing the trigone with a 30-gauge needle connected to a microdialysis pump (1 µL/min; CMA/102; Microdialysis AB, Solna, Sweden) [18,19]. A total volume of 12.5 µL was evenly distributed in four divided sites for the S-4 sites and *aboBoNTA*-4 sites (5.6 U in 3.125 µL *i.e.*, 1.8 U/µL per site) groups and a total volume of 25 µL in eight divided sites for S-8 sites and *aboBoNTA*-8 sites (2.8 U in 3.125 µL *i.e.*, 0.9 U/µL per site) groups (Scheme 1). A PE-50 catheter was inserted within the bladder dome, tunnelled subcutaneously, exteriorized at the back of the neck, and sutured between the scapulae. Postoperatively, rats were treated with 10 mg/kg gentamicin.

### 5.3. Cystometry Experiments in Conscious Spinal-Cord-Injured Rats

Cystometry was performed in conscious rats 48 h after intradetrusor injections, as previously described [21]. Clinically-relevant voiding and filling parameters were analyzed: maximal pressure of voiding contractions (VC) (millimetre of mercury, mmHg; 1 mmHg corresponding to 1.36 cm·H_2_O), infused volume (microliters, µL) as an index of bladder capacity, compliance (mL per mmHg) and voiding efficiency (%, ratio of voided volume to infused volume), as residual volume is not measurable during continuous cystometry. Clinically-relevant parameters characterizing bladder nonvoiding contractions (NVC), defined as contractions with an amplitude of ≥ 3 mmHg during the filling phase, were analyzed: amplitude of NVC (mmHg), frequency of NVC (number per min) and volume threshold to elicit NVC (% of total bladder-filling volume), which is the preclinical equivalent to the volume at first involuntary detrusor contraction in clinical urodynamic investigations [10,41].

### 5.4. Statistical Analysis

All data were expressed as mean plus or minus the standard error of the mean and averaged per treatment group. A Grubbs’ test was used for exclusion of outliers. An unpaired Student’s *t*-test was used for the comparison of body weight (GraphPad Prism^®^ 5.04, La Jolla, CA, USA, 2010). The area under curve (AUC) of BWL was compared between aboBoNTA-4 sites or aboBoNTA-8 sites groups for homogeneity with Fisher’s test and for significance with Student’s *t*-test (SAS^®^ USA version 9.2, Cary, NC, USA, 2009). For each cystometry parameter, an analysis of variance was performed to compare (1) the S-4 sites and S-8 sites saline groups to allow their aggregation, and (2) the aboBoNTA-4 sites or aboBoNTA-8 sites groups *versus* the aggregated saline groups (SAS^®^). *p* values < 0.05 were considered significant.

### 5.5. Drugs and Chemicals

*AboBoNTA* (*Batch E194441*) was provided by Ipsen Biopharm Ltd., Wrexham, UK. Antibiotics (except gentamicin) and anaesthetics were purchased from Centravet (Dinan, France) and Roche Pharma (Neuilly-sur-Seine, France). Gentamicin was purchased from Merck and Co. Inc. (Whitehouse Station, NJ, USA). All other drugs and chemicals were purchased from Sigma-Aldrich (Lyon, France).

## References

[1] Schurch B., Stohrer M., Kramer G., Schmid D.M., Gaul G., Hauri D. (2000). *Botulinum*-A toxin for treating detrusor hyperreflexia in spinal cord injured patients: A new alternative to anticholinergic drugs? Preliminary results. J. Urol..

[2] Smith C.P., Franks M.E., McNeil B.K., Ghosh R., de Groat W.C., Chancellor M.B., Somogyi G.T. (2003). Effect of *botulinum toxin A* on the autonomic nervous system of the rat lower urinary tract. J. Urol..

[3] Khera M., Somogyi G.T., Kiss S., Boone T.B., Smith C.P. (2004). *Botulinum* toxin A inhibits ATP release from bladder urothelium after chronic spinal cord injury. Neurochem. Int..

[4] Liu H.T., Chancellor M.B., Kuo H.C. (2009). Urinary nerve growth factor levels are elevated in patients with detrusor overactivity and decreased in responders to detrusor *botulinum* toxin-A injection. Eur. Urol..

[5] Smith C.P., Boone T.B., de Groat W.C., Chancellor M.B., Somogyi G.T. (2003). Effect of stimulation intensity and *botulinum* toxin isoform on rat bladder strip contractions. Brain Res. Bull..

[6] Apostolidis A., Dasgupta P., Fowler C.J. (2006). Proposed mechanism for the efficacy of injected *botulinum* toxin in the treatment of human detrusor overactivity. Eur. Urol..

[7] Munoz A., Somogyi G.T., Boone T.B., Smith C.P. (2011). Central inhibitory effect of intravesically applied *botulinum* toxin A in chronic spinal cord injury. Neurourol. Urodyn..

[8] Kanai A., Wyndaele J.J., Andersson K.E., Fry C., Ikeda Y., Zabbarova I., De W.S. (2011). Researching bladder afferents—determining the effects of β_3_-adrenergic receptor agonists and *botulinum* toxin type-A. Neurourol. Urodyn..

[9] Chuang Y.C., Tyagi P., Huang C.C., Yoshimura N., Wu M., Kaufman J., Chancellor M.B. (2009). Urodynamic and immunohistochemical evaluation of intravesical *botulinum* toxin A delivery using liposomes. J. Urol..

[10] Cruz F., Herschorn S., Aliotta P., Brin M., Thompson C., Lam W., Daniell G., Heesakkers J., Haag-Molkenteller C. (2011). Efficacy and safety of *onabotulinumtoxinA* in patients with urinary incontinence due to neurogenic detrusor overactivity: A randomised, double-blind, placebo-controlled trial. Eur. Urol..

[11] Ehren I., Volz D., Farrelly E., Berglund L., Brundin L., Hultling C., Lafolie P. (2007). Efficacy and impact of *botulinum* toxin A on quality of life in patients with neurogenic detrusor overactivity: A randomised, placebo-controlled, double-blind study. Scand. J. Urol. Nephrol..

[12] Ginsberg D., Gousse A., Keppenne V., Sievert K.D., Thompson C., Lam W., Brin M.F., Jenkins B., Haag-Molkenteller C. (2012). Phase 3 efficacy and tolerability study of *onabotulinumtoxinA* for urinary incontinence from neurogenic detrusor overactivity. J. Urol..

[13] Herschorn S., Gajewski J., Ethans K., Corcos J., Carlson K., Bailly G., Bard R., Valiquette L., Baverstock R., Carr L., Radomski S. (2011). Efficacy of *botulinum* toxin A injection for neurogenic detrusor overactivity and urinary incontinence: A randomized, double-blind trial. J. Urol..

[14] Rovner E., Dmochowski R., Chapple C., Thompson C., Lam W., Haag-Molkenteller C. (2013). *OnabotulinumtoxinA* improves urodynamic outcomes in patients with neurogenic detrusor overactivity. Neurourol. Urodyn..

[15] Schurch B., de Seze M., Denys P., Chartier-Kastler E., Haab F., Everaert K., Plante P., Perrouin-Verbe B., Kumar C., Fraczek S., Brin M.F. (2005). *Botulinum* toxin type a is a safe and effective treatment for neurogenic urinary incontinence: Results of a single treatment, randomized, placebo controlled 6-month study. J. Urol..

[16] Mangera A., Andersson K.E., Apostolidis A., Chapple C., Dasgupta P., Giannantoni A., Gravas S., Madersbacher S. (2011). Contemporary management of lower urinary tract disease with botulinum toxin A: A systematic review of botox (*onabotulinumtoxinA*) and dysport (*abobotulinumtoxinA*). Eur. Urol..

[17] Shakeri S., Mohammadian R., Aminsharifi A., Ariafar A., Vaghedashti J., Yazdani M., Yadollahi M., Emadmarvasti V., Baharikhoob A. (2014). Success rate and patients' satisfaction following intradetrusor dysport injection in patients with detrusor overactivity: A comparative study of idiopathic and neurogenic types of detrusor overactivity. Urol. J..

[18] Smith C.P., Gangitano D.A., Munoz A., Salas N.A., Boone T.B., Aoki K.R., Francis J., Somogyi G.T. (2008). *Botulinum* toxin type A normalizes alterations in urothelial ATP and NO release induced by chronic spinal cord injury. Neurochem. Int..

[19] Temeltas G., Tikiz C., Dagci T., Tuglu I., Yavasoglu A. (2005). The effects of *botulinum*-A toxin on bladder function and histology in spinal cord injured rats: Is there any difference between early and late application?. J. Urol..

[20] Leung P.Y., Johnson C.S., Wrathall J.R. (2007). Comparison of the effects of complete and incomplete spinal cord injury on lower urinary tract function as evaluated in unanesthetized rats. Exp. Neurol..

[21] Behr-Roussel D., Oger S., Pignol B., Pham E., Le M.A., Chabrier P.E., Caisey S., Compagnie S., Picaut P., Bernabe J. (2012). Minimal effective dose of dysport and botox in a rat model of neurogenic detrusor overactivity. Eur. Urol..

[22] Cheng C.L., de Groat W.C. (2004). The role of capsaicin-sensitive afferent fibers in the lower urinary tract dysfunction induced by chronic spinal cord injury in rats. Exp. Neurol..

[23] Grosse J., Kramer G., Jakse G. (2009). Comparing two types of *botulinum*-A toxin detrusor injections in patients with severe neurogenic detrusor overactivity: A case-control study. BJU Int..

[24] Wenzel R., Jones D., Borrego J.A. (2007). Comparing two *botulinum* toxin type A formulations using manufacturers’ product summaries. J. Clin. Pharm. Ther..

[25] Linsenmeyer T.A. (2013). Use of botulinum toxin in individuals with neurogenic detrusor overactivity: State of the art review. J. Spin. Cord Med..

[26] Karsenty G., Denys P., Amarenco G., de Seze M., Game X., Haab F., Kerdraon J., Perrouin-Verbe B., Ruffion A., Saussine C. (2008). *Botulinum* toxin A (Botox) intradetrusor injections in adults with neurogenic detrusor overactivity/neurogenic overactive bladder: A systematic literature review. Eur. Urol..

[27] Cruz F. (2014). Targets for *botulinum* toxin in the lower urinary tract. Neurourol. Urodyn..

[28] Apostolidis A., Popat R., Yiangou Y., Cockayne D., Ford A.P., Davis J.B., Dasgupta P., Fowler C.J., Anand P. (2005). Decreased sensory receptors P2X3 and TRPV1 in suburothelial nerve fibers following intradetrusor injections of *botulinum* toxin for human detrusor overactivity. J. Urol..

[29] Giannantoni A., di Stasi S.M., Nardicchi V., Zucchi A., Macchioni L., Bini V., Goracci G., Porena M. (2006). *Botulinum*-A toxin injections into the detrusor muscle decrease nerve growth factor bladder tissue levels in patients with neurogenic detrusor overactivity. J. Urol..

[30] Giannantoni A., Conte A., Farfariello V., Proietti S., Vianello A., Nardicchi V., Santoni G., Amantini C. (2013). *Onabotulinumtoxin*-A intradetrusorial injections modulate bladder expression of NGF, TrkA, p75 and TRPV1 in patients with detrusor overactivity. Pharmacol. Res..

[31] Takahashi R., Yunoki T., Naito S., Yoshimura N. (2012). Differential effects of *botulinum* neurotoxin A on bladder contractile responses to activation of efferent nerves, smooth muscles and afferent nerves in rats. J. Urol..

[32] Mehnert U., Boy S., Schmid M., Reitz A., von H.A., Hodler J., Schurch B. (2009). A morphological evaluation of *botulinum* neurotoxin A injections into the detrusor muscle using magnetic resonance imaging. World J. Urol..

[33] Coelho A., Cruz F., Cruz C.D., Avelino A. (2012). Spread of *onabotulinumtoxinA* after bladder injection. Experimental study using the distribution of cleaved SNAP-25 as the marker of the toxin action. Eur. Urol..

[34] Karsenty G., Carsenac A., Boy S., Reitz A., Bardot P., Tournebise H. (2007). *Botulinum* toxin-A (BTA) in the treatmentof neurogenic detrusor overactivity incontinence (NDOI)d a prospective randomized study to compare 30 *vs.* 10 injection sites (abstract 890). Eur. Urol..

[35] Liao C.H., Chen S.F., Kuo H.C. (2015). Different number of intravesical *onabotulinumtoxinA* injections for patients with refractory detrusor overactivity do not affect treatment outcome: A prospective randomized comparative study. Neurourol. Urodyn..

[36] Ramirez-Castaneda J., Jankovic J., Comella C., Dashtipour K., Fernandez H.H., Mari Z. (2013). Diffusion, spread, and migration of *botulinum* toxin. Mov. Disord..

[37] Stone H.F., Zhu Z., Thach T.Q., Ruegg C.L. (2011). Characterization of diffusion and duration of action of a new *botulinum* toxin type A formulation. Toxicon.

[38] Coskun H., Duran Y., Dilege E., Mihmanli M., Seymen H., Demirkol M.O. (2005). Effect on gastric emptying and weight reduction of *botulinum* toxin-A injection into the gastric antral layer: An experimental study in the obese rat model. Obes. Surg..

[39] Del Popolo G. (2001). *Botulinum*-A toxin in the treatment of detrusor hyperreflexia. Neurourol. Urodyn..

[40] Kuo H.C. (2004). Urodynamic evidence of effectiveness of *botulinum* A toxin injection in treatment of detrusor overactivity refractory to anticholinergic agents. Urology.

[41] Grise P., Ruffion A., Denys P., Egon G., Chartier K.E. (2010). Efficacy and tolerability of *botulinum* toxin type A in patients with neurogenic detrusor overactivity and without concomitant anticholinergic therapy: Comparison of two doses. Eur. Urol..

[42] De Laet K., Wyndaele J.J. (2005). Adverse events after *botulinum* A toxin injection for neurogenic voiding disorders. Spin. Cord.

